# Academic Self-Handicapping, Buoyancy, and Burnout in Junior High School: Longitudinal Dynamics with Implications for School-Based Prevention

**DOI:** 10.3390/bs16050780

**Published:** 2026-05-14

**Authors:** Licong Ye, Zipiao Zhang, Baojuan Ye, Bin Zhou

**Affiliations:** 1 Public Teaching Department, Fuzhou Medical College, Fuzhou 344000, China; 2 School of Psychology, Jiangxi Normal University, Nanchang 330022, China

**Keywords:** academic self-handicapping, academic buoyancy, academic burnout, coping, protective factors, risk markers, early adolescence, school-based mental health, longitudinal design, CLPM

## Abstract

This study examined the longitudinal associations among academic self-handicapping, academic buoyancy, and academic burnout, and tested whether academic buoyancy played a mediating role in the association between academic self-handicapping and academic burnout. A three-wave longitudinal survey with 3-month intervals was conducted among 508 Chinese junior high school students (Grades 7–9; Mage = 13.44 years; 48.8% boys). Cross-lagged panel modeling (CLPM) was used for data analysis. Results indicated that (1) academic self-handicapping and academic buoyancy showed reciprocal negative longitudinal associations; (2) academic buoyancy and academic burnout also showed reciprocal negative longitudinal associations; and (3) academic buoyancy showed a nuanced longitudinal mediating pattern: the hypothesized indirect effect from academic self-handicapping to academic burnout was marginally significant, whereas the reverse indirect effect was significant. From a school-based mental health and prevention perspective, these findings highlight academic buoyancy as a modifiable protective resource and academic self-handicapping as a potentially observable coping-related risk marker, suggesting actionable targets for early identification and tiered support to mitigate burnout-related disengagement in junior high school students.

## 1. Introduction

Academic burnout typically manifests as persistent exhaustion, growing detachment from schoolwork, and diminished feelings of accomplishment in the learning domain (Maslach et al., 2001; Schaufeli et al., 2002). It is a nontrivial concern among young adolescents, and prevalence estimates vary substantially across studies; a recent synthesis of 11 studies reported an average prevalence of 14.36%, whereas one study identified 32.2% of participants as fitting a burnout profile (Lacombe et al., 2023). In many exam-oriented educational contexts, frequent examinations and salient performance evaluation are routine, and repeated setbacks and sustained pressure may therefore accumulate into academic burnout. Such burnout can have far-reaching consequences, including persistent erosion of learning engagement and achievement, heightened vulnerability to psychological distress, and spillover risks for longer-term developmental maladjustment (Fiorilli et al., 2017; Gao, 2023; Wu et al., 2007). Accordingly, academic burnout is increasingly relevant not only to educational outcomes but also to school-based mental health and prevention efforts. These concerns highlight the need to identify modifiable risk and protective processes that shape how academic burnout emerges and unfolds during early adolescence, thereby informing more targeted efforts to support adolescents’ healthy development within schools, including early identification and preventive support across educational, family, and broader social contexts.

One underexamined proximal risk process for adolescent academic burnout is academic self-handicapping, yet research in junior high school students is still limited and largely cross-sectional (Khurshid et al., 2025; H. Zhao et al., 2024). However, this pattern is noteworthy because self-handicapping aims to shield the self under performance threat but may simultaneously undermine engagement and amplify pressure—conditions that foster burnout (Midgley et al., 1996; Schaufeli et al., 2002). At the heart of this work is a process account that considers self-handicapping alongside academic buoyancy, an everyday resilience resource for coping with routine academic setbacks (Martin et al., 2013; Martin & Marsh, 2008). Rather than treating buoyancy as a static correlate, we position it as a key resource that may help explain the association between self-handicapping and burnout (Lakkavaara et al., 2024; Martin et al., 2010; Martin & Marsh, 2020). From a prevention standpoint, this framing highlights self-handicapping as a coping-related risk process and buoyancy as a potentially modifiable protective resource that schools can target to reduce burnout-related disengagement.

In making this argument, we draw on self-worth theory to conceptualize academic self-handicapping as a self-protective strategy enacted when academic performance is treated as a central indicator of ability and self-esteem (Covington, 1992). Specifically, to pre-empt the threat of failure, students may withdraw effort, procrastinate, or downplay task importance so that poor outcomes can be attributed to external impediments rather than low ability (Berglas & Jones, 1978; Midgley et al., 1996; Saltürk, 2022). Such strategies may diminish perceived control and heighten academic anxiety, thereby undermining academic buoyancy—students’ capacity to bounce back from everyday academic setbacks (Martin, 2013; Martin & Marsh, 2020). From the motivational resilience and vulnerability model, buoyancy reflects adaptive coping in routine academic adversity; lower buoyancy should therefore be accompanied by more maladaptive coping and greater exhaustion and disengagement, aligning with core features of academic burnout (Schaufeli et al., 2002; F. Zhao & Yu, 2018). Thus, buoyancy is positioned as a theoretically grounded protective resource that may help explain the association between self-handicapping and academic burnout during early adolescence. Taken together, these considerations motivate a mechanism-focused account centered on academic buoyancy to clarify the self-handicapping–burnout association.

### 1.1. Academic Self-Handicapping, Academic Buoyancy, and Self-Worth Processes

Academic buoyancy refers to students’ capacity to bounce back from routine academic setbacks and to sustain adaptive coping under everyday academic pressure (Martin & Marsh, 2008). From a self-worth perspective, academic self-handicapping—such as procrastination, effort withdrawal, or downplaying task importance—functions as a short-term strategy to protect self-esteem when performance is threatening (Covington, 1992; Midgley et al., 1996). Yet such strategies may carry longer-term costs for coping resources. By shifting attention away from constructive effort and toward excuse-based regulation, self-handicapping can erode students’ perceived control over learning outcomes and heighten academic anxiety, conditions that are inconsistent with buoyancy and may drain the psychological energy needed to “bounce back” after setbacks (Martin & Marsh, 2008). Consistent with this account, empirical work has found that higher self-handicapping is associated with lower buoyancy (Jia et al., 2020). The reverse association is also theoretically plausible. Students with higher academic buoyancy tend to sustain problem-focused coping and persistence in the face of everyday academic setbacks, potentially lowering their dependence on avoidant self-protective strategies such as self-handicapping. This proposition is consistent with scholarship framing academic buoyancy as adaptive coping with routine academic adversity and with work linking avoidance-oriented responses to maladaptive academic functioning under stress (Martin, 2013; Putwain et al., 2023). Overall, prior evidence and theory suggest that this association is likely negative, with greater self-handicapping accompanying lower buoyancy and higher buoyancy accompanying less self-handicapping. Accordingly, we hypothesize that academic self-handicapping and academic buoyancy show a reciprocal association (H1).

### 1.2. Academic Buoyancy, Academic Burnout, and Resource-Based Processes

Academic buoyancy is widely viewed as a protective personal resource in students’ everyday academic lives, enabling them to recover from routine setbacks while maintaining motivation and adaptive coping (Martin & Marsh, 2008). Accordingly, students higher in buoyancy tend to show lower levels of academic burnout, including reduced exhaustion, less disengagement, and fewer feelings of low accomplishment (Collie et al., 2015; Putwain et al., 2022). Buoyancy may also shape how academic stress and negative emotions translate into maladjustment: by sustaining a sense of control and persistence under pressure, buoyant students are less likely to accumulate the prolonged strain that characterizes burnout (Anyan & Hjemdal, 2016; Putwain et al., 2022). At the same time, burnout may be associated with diminished buoyancy. Exhaustion can drain the psychological energy needed for emotion regulation and perseverance; disengagement can weaken motivation for adaptive coping; and reduced accomplishment can erode efficacy-related beliefs, together undermining the resources that support “bouncing back” in daily academic contexts (Collie et al., 2015; Martin & Marsh, 2008). Accordingly, we hypothesize that academic buoyancy and academic burnout show a reciprocal association (H2).

### 1.3. The Proposed Process Model Linking Academic Self-Handicapping, Academic Buoyancy, and Academic Burnout

Building on self-worth theory and the motivational resilience and vulnerability model, we propose academic buoyancy as a plausible mechanism that helps explain why academic self-handicapping may be associated with academic burnout. From a self-worth perspective, self-handicapping is often used to protect self-esteem under evaluative threat, for example by procrastinating, withdrawing effort, or downplaying task importance (Covington, 1992; Midgley et al., 1996). Although such strategies may be self-protective in the short term, they can also undermine students’ perceived control and shift coping away from constructive engagement, thereby weakening the adaptive resources needed to deal with everyday academic setbacks (Martin & Marsh, 2008). From this perspective, a negative association between self-handicapping and academic buoyancy is theoretically plausible, but direct evidence remains limited; thus, further testing is needed, particularly in adolescent samples (Jia et al., 2020).

From the motivational resilience and vulnerability model, buoyancy reflects adaptive coping and emotion-regulation processes that enable students to persist through routine academic adversity (Martin & Marsh, 2008; F. Zhao & Yu, 2018). Higher buoyancy has been associated with lower academic burnout and related maladjustment, suggesting that buoyancy may function as a protective resource that buffers daily academic strain before it consolidates into more chronic exhaustion and disengagement (Collie et al., 2015; Lakkavaara et al., 2024; Putwain et al., 2022). Taken together, these strands point to a coherent process account: self-handicapping may be associated with lower buoyancy, and reduced buoyancy may in turn be associated with greater burnout risk. Because directionality cannot be inferred from cross-sectional evidence alone, multi-wave longitudinal designs are needed to clarify temporal ordering and to evaluate the proposed mechanism within a dynamic system (Adachi & Willoughby, 2015). Accordingly, we hypothesize that academic buoyancy plays a mediating role in the association between academic self-handicapping and academic burnout (H3).

Early adolescence is a period when students’ academic motivation and coping resources are especially malleable, yet also vulnerable to cumulative evaluative demands. Exam-oriented schooling in high-stakes contexts, including China, provides a stringent context in which to examine the proposed process model, while the focal mechanisms—academic self-handicapping, academic buoyancy, and academic burnout—remain relevant for adolescents facing comparable evaluative demands across education systems. In this broader context, academic buoyancy—the capacity to rebound from routine setbacks—represents a potentially modifiable resource that may interrupt pathways from short-term self-protection to longer-term exhaustion and disengagement. These considerations underscore the value of a mechanism-focused account of academic burnout during early adolescence.

In sum, the present study aims to examine the longitudinal associations among academic self-handicapping, academic buoyancy, and academic burnout, and to reveal the potential mediating role of academic buoyancy in these associations. By clarifying the temporal ordering and dynamic coupling among a coping-related risk behavior (self-handicapping), an everyday resilience-related protective resource (buoyancy), and a school-adjustment risk outcome (burnout), the study is intended to inform school-based mental health and prevention efforts, including early identification and tiered support in junior high school settings. Specifically, we investigate: (1) the reciprocal association between academic self-handicapping and academic buoyancy; (2) the reciprocal association between academic buoyancy and academic burnout; and (3) whether academic buoyancy plays a mediating role in the association between academic self-handicapping and academic burnout.

## 2. Materials and Methods

### 2.1. Participants

Participants were junior high school students (Grades 7–9) in China. A random classroom-based sampling approach was used, with intact classes selected from each grade level. Because students were surveyed within naturally occurring classrooms, the final sample had a clustered structure, with students nested within 16 classrooms. After matching cases across the three survey waves and removing invalid questionnaires, the final analytic sample comprised 508 students, including 248 boys (48.8%) and 260 girls (51.2%), with a mean age of 13.44 years (SD = 0.97). The sample included 185 Grade 7 students (36.4%), 145 Grade 8 students (28.5%), and 178 Grade 9 students (35.0%). Regarding family structure, 30 students (5.9%) were only children and 478 (94.1%) were non-only children. With respect to place of origin, 171 students (33.7%) were from urban areas and 337 (66.3%) were from rural areas. All participants completed questionnaires in classroom settings with assurances of confidentiality and voluntary participation.

### 2.2. Research Tools

#### 2.2.1. Academic Self-Handicapping Questionnaire

Academic self-handicapping was assessed using the Academic Self-Handicapping Questionnaire for Middle School Students (Huang, 2006). The questionnaire contains 15 items and comprises two dimensions: behavioral self-handicapping and claimed self-handicapping. Items were rated on a 5-point Likert scale ranging from 1 (strongly disagree) to 5 (strongly agree), with higher scores indicating higher levels of academic self-handicapping. In the present study, Cronbach’s α values across the three waves were 0.817 (T1), 0.906 (T2), and 0.942 (T3).

#### 2.2.2. Academic Buoyancy Scale

Academic buoyancy was measured using the Academic Buoyancy Scale (Martin & Marsh, 2008). The scale consists of 4 items rated on a 7-point Likert scale ranging from 1 (strongly disagree) to 7 (strongly agree), with higher scores indicating higher levels of academic buoyancy. In the present study, Cronbach’s α values across the three waves were 0.812 (T1), 0.797 (T2), and 0.825 (T3).

#### 2.2.3. Academic Burnout Scale

Academic burnout was assessed using the Adolescent Academic Burnout Scale (Wu et al., 2007). The scale includes 16 items covering three dimensions: physical–mental exhaustion, academic alienation, and reduced sense of accomplishment. Items were rated on a 5-point Likert scale ranging from 1 (strongly disagree) to 5 (strongly agree), with higher scores indicating more severe academic burnout. In the present study, Cronbach’s α values across the three waves were 0.796 (T1), 0.782 (T2), and 0.797 (T3).

#### 2.2.4. Control Variables

Given that adolescents’ gender and grade level may be associated with academic self-handicapping, academic buoyancy, and academic burnout (Li et al., 2021), gender and grade were included as covariates in the analyses.

### 2.3. Research Procedure

Ethical review and approval were waived for this study by the Medical Ethics Review Committee, Fuzhou Medical College, Nanchang University, because the study involved a minimal-risk questionnaire survey conducted in a normal educational setting and met the criteria for exemption review. Prior to administration, permission was obtained from the participating school(s), and written informed consent was obtained from students and their parents/guardians. After participants were informed of the study purpose, a school psychologist served as the administrator and organized the assessment by class in a standard multimedia classroom. Students completed the Academic Self-Handicapping Questionnaire, the Academic Buoyancy Scale, and the Academic Burnout Scale using paper-and-pencil formats. Confidentiality was emphasized, and students were reminded that there were no “right or wrong” answers and that they should respond honestly. Each assessment took approximately 25 min, and questionnaires were collected immediately upon completion. The same procedure was used across all three waves.

### 2.4. Data Analysis

First, Mplus 8.11 was used to conduct longitudinal confirmatory factor analyses (CFA) to test measurement invariance of academic self-handicapping, academic buoyancy, and academic burnout across the three waves. We sequentially specified a configural invariance model (baseline), a metric invariance model (constraining factor loadings to be equal across time), and a scalar invariance model (further constraining item intercepts to be equal across time) (Meade et al., 2005). Measurement invariance was evaluated using changes in model fit; when the decrease in CFI was less than 0.01, invariance across time was considered acceptable. Second, SPSS 26.0 was used to compute descriptive statistics and Pearson correlations among the study variables. Third, cross-lagged panel models (CLPMs) were estimated in Mplus to examine the longitudinal associations among academic self-handicapping, academic buoyancy, and academic burnout, with gender and grade included as covariates. Given the classroom-based sampling design, students were nested within classrooms. To account for the non-independence of observations, the CLPM analyses were estimated using the TYPE = COMPLEX option in Mplus, with the grade-class combined classroom identifier specified as the clustering variable, thereby producing cluster-robust standard errors. Missing values were handled using full information maximum likelihood (FIML) estimation in Mplus. Unless otherwise noted, path coefficients reported in the CLPM are standardized estimates and are denoted by *γ*. Indirect effects are reported as standardized indirect effects based on the STDYX standardized results from Mplus. All tests were two-tailed, with *p* < 0.05 indicating statistical significance.

## 3. Results

### 3.1. Measurement Equivalence Test

To ensure that academic self-handicapping, academic buoyancy, and academic burnout were comparable across the three waves, we conducted longitudinal confirmatory factor analyses in Mplus 8.11 and tested configuration, weak, and strong measurement equivalence. Following established guidelines, measurement equivalence was supported when changes in fit indices met the criteria of ΔCFI ≤ 0.01 and ΔRMSEA ≤ 0.015, indicating that imposing more restrictive equality constraints did not substantially worsen model fit (Chen, 2007). As shown in Table 1, the academic buoyancy and academic burnout measures supported strong equivalence across waves. For academic self-handicapping, the strong-equivalence model yielded a ΔCFI that slightly exceeded the recommended cutoff, whereas ΔRMSEA remained within the guideline, suggesting that equivalence for this construct should be interpreted cautiously.

### 3.2. Descriptive Statistics and Correlation Analysis

Correlations among academic self-handicapping, academic buoyancy, and academic burnout across the three waves are shown in Table 2. Across waves, academic self-handicapping was significantly positively associated with academic burnout and significantly negatively associated with academic buoyancy, whereas academic buoyancy was significantly negatively associated with academic burnout.

Based on the guidelines for interpreting correlation coefficients (Cohen, 1992) where |*r*| ≥ 0.10 is small, |*r*| ≥ 0.30 is medium, and |*r*| ≥ 0.50 is large, the stabilities and concurrent correlations in the present study demonstrated medium to large effect sizes. As shown in Table 2, the longitudinal stabilities were substantial, such as the association between T1 and T2 academic self-handicapping (*r* = 0.56), between T1 and T2 academic buoyancy (*r* = 0.59), and between T1 and T2 academic burnout (*r* = 0.76); similarly, the stability from T2 to T3 was also evident for self-handicapping (*r* = 0.53), buoyancy (*r* = 0.58), and burnout (*r* = 0.70). Concurrently, the correlations were also meaningful, including the negative associations between academic buoyancy and academic burnout at each wave (T1: *r* = −0.51; T2: *r* = −0.43; T3: *r* = −0.49). These findings confirm that the variables are not only statistically associated but also show meaningful associations, providing a solid foundation for the cross-lagged analyses.

### 3.3. Cross-Lagged Associations Among Academic Self-Handicapping, Academic Buoyancy, and Academic Burnout

**Figure 1:**
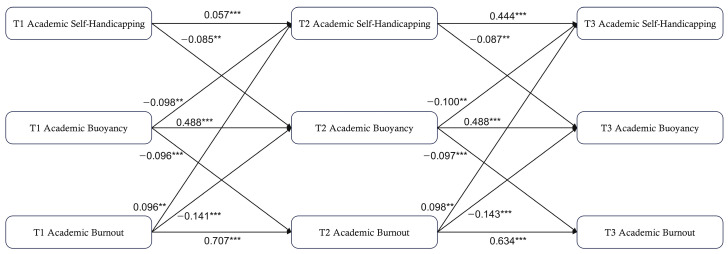
Cross-lagged model diagram of Academic Self-Handicapping, Academic Buoyancy, and Academic Burnout. Values shown are standardized path coefficients, denoted as *γ* in the text. For clarity, autoregressive paths, within-wave correlations, covariates, and nonsignificant paths are omitted. ** *p* < 0.01, *** *p* < 0.001. Standardized indirect effects and their 95% confidence intervals are reported in Table 3.

In the cross-lagged panel model, gender and grade were included as covariates given their potential associations with academic self-handicapping, academic buoyancy, and academic burnout. The model was estimated with cluster-robust standard errors to account for the nesting of students within classrooms. The model showed acceptable fit to the data, *CFI* = 0.986, *TLI* = 0.950, *RMSEA* = 0.057, and *SRMR* = 0.022. As shown in Figure 1, academic self-handicapping showed significant negative cross-lagged associations with subsequent academic buoyancy from T1 to T2 (*γ* = −0.085, *t* = −3.179, *p* < 0.01) and from T2 to T3 (*γ* = −0.087, *t* = −3.233, *p* < 0.01). Conversely, academic buoyancy showed significant negative cross-lagged associations with subsequent academic self-handicapping from T1 to T2 (*γ* = −0.098, *t* = −3.117, *p* < 0.01) and from T2 to T3 (*γ* = −0.100, *t* = −3.143, *p* < 0.01), indicating reciprocal associations between self-handicapping and buoyancy.

Meanwhile, academic buoyancy and academic burnout also exhibited reciprocal negative cross-lagged associations. Academic buoyancy showed significant negative cross-lagged associations with subsequent academic burnout from T1 to T2 (γ = −0.096, *t* = −3.547, *p* < 0.01) and from T2 to T3 (*γ* = −0.097, *t* = −3.449, *p* < 0.01). In the reverse direction, academic burnout showed significant negative cross-lagged associations with subsequent academic buoyancy from T1 to T2 (*γ* = −0.141, *t* = −4.681, *p* < 0.001) and from T2 to T3 (*γ* = −0.143, *t* = −4.732, *p* < 0.001).

For the association between academic self-handicapping and academic burnout, the cross-lagged paths from self-handicapping to later burnout were not significant from T1 to T2 (*γ* = 0.040, *t* = 1.609, *p* > 0.05) or from T2 to T3 (*γ* = 0.041, *t* = 1.628, *p* > 0.05). However, academic burnout showed significant positive cross-lagged associations with later academic self-handicapping from T1 to T2 (*γ* = 0.096, *t* = 2.986, *p* < 0.01) and from T2 to T3 (*γ* = 0.098, *t* = 2.867, *p* < 0.01).

Overall, the standardized cross-lagged coefficients were small in magnitude. These effects should therefore be interpreted cautiously and in the context of an autoregressive longitudinal model, in which each construct was predicted after controlling for its prior level. From this perspective, the findings indicate modest but consistent prospective associations with residual change rather than large effects on later absolute levels.

To test the indirect effect, the standardized indirect effects were estimated based on the STDYX standardized results from Mplus. After adjusting for classroom clustering, the indirect effect from academic self-handicapping to academic burnout via academic buoyancy was in the expected direction and marginally significant (standardized indirect effect = 0.008, *p* = 0.059, 95% CI [0.000, 0.017]). In addition, a significant reverse standardized indirect effect was observed from academic burnout to subsequent academic self-handicapping via academic buoyancy (standardized indirect effect = 0.014, *p* = 0.014, 95% CI [0.003, 0.025]). To improve clarity and to map the findings onto the research questions, the key standardized cross-lagged paths and standardized indirect effects are summarized in Table 3.

## 4. Discussion

### 4.1. Academic Self-Handicapping and Academic Buoyancy

Consistent with H1, academic self-handicapping and academic buoyancy showed a reciprocal negative association over time. This pattern aligns with self-worth theory (Covington, 1992), which conceptualizes self-handicapping as a short-term self-protective tactic enacted under evaluative threat. Although procrastination, effort withdrawal, and excuse-making may temporarily buffer self-esteem, these tactics tend to shift students away from constructive engagement and toward avoidant regulation, thereby undermining perceived control and increasing academic anxiety. Such costs are incompatible with buoyancy, which depends on sustaining adaptive coping and rapid recovery in the face of routine setbacks (Martin & Marsh, 2008). In this sense, self-handicapping may gradually “tax” the coping resources that buoyancy represents, making it harder for students to rebound effectively following everyday academic difficulties (Jia et al., 2020; Sun, 2023). Importantly, the longitudinal pattern observed here suggests that these costs may accumulate over time rather than remain confined to single episodes of academic stress.

The reverse association is also theoretically plausible. Buoyant students are more likely to persist, reappraise setbacks, and use problem-focused coping in daily academic challenges, which reduces the need to pre-empt failure through self-protective avoidance (Martin & Marsh, 2008). Prior evidence has suggested a negative association between academic buoyancy and self-handicapping, but direct evidence remains limited, particularly in adolescent samples (Jia et al., 2020). Taken together, the present findings extend emerging work by indicating that self-handicapping and buoyancy may form a mutually reinforcing cycle in early adolescence: self-handicapping can erode buoyancy, while buoyancy can function as a protective resource that helps curb reliance on self-handicapping.

### 4.2. Academic Buoyancy and Academic Burnout

Consistent with H2, the present study found that academic buoyancy and academic burnout showed a reciprocal negative association over time. From the motivational resilience and vulnerability perspective, buoyancy reflects students’ capacity for adaptive coping in routine academic adversity; as such, it functions as a protective resource that can curb the accumulation of strain into more chronic exhaustion and disengagement (Martin & Marsh, 2008; F. Zhao & Yu, 2018). Empirically, higher buoyancy has been associated with lower burnout and related maladjustment, suggesting that buoyancy helps sustain control, persistence, and emotion regulation under everyday setbacks (Anyan & Hjemdal, 2016; Collie et al., 2015; Putwain et al., 2022).

Meanwhile, the reverse association is also theoretically expected. Academic burnout is characterized by emotional exhaustion, academic alienation, and reduced accomplishment, which may progressively erode the psychological energy, motivational engagement, and efficacy-related beliefs needed to “bounce back” when difficulties arise (Martin & Marsh, 2008; Schaufeli et al., 2002; Wu et al., 2007). Taken together, the findings suggest that buoyancy and burnout may co-develop in a mutually reinforcing way, highlighting buoyancy as a salient leverage point for interrupting early burnout trajectories (Putwain et al., 2022; Xu, 2023).

### 4.3. Academic Buoyancy as a Mediator Between Academic Self-Handicapping and Academic Burnout

Partially consistent with H3, the present longitudinal study showed that the indirect effect from academic self-handicapping to academic burnout via academic buoyancy was in the expected direction and marginally significant after adjusting for classroom clustering. Specifically, higher self-handicapping at an earlier time point tended to be with lower subsequent buoyancy, which in turn tended to be associated with higher subsequent burnout. This pattern is consistent with accumulating evidence that self-handicapping is negatively associated with buoyancy (Jia et al., 2020; Sun, 2023) and that depleted buoyancy may undermine adaptive academic engagement, thereby increasing vulnerability to burnout over time (Collie et al., 2015; Putwain et al., 2022). Importantly, the present findings extend these pairwise associations by providing cautious support for a longitudinal process account in which academic buoyancy may function as a resource-related process linking self-protective self-handicapping to later academic burnout.

From a self-worth perspective, self-handicapping functions as a short-term self-protective maneuver under evaluative threat, yet it can entail longer-term psychological costs (Covington, 1992; Midgley et al., 1996). When students procrastinate, withdraw effort, or downplay task importance, these strategies may reduce perceived control and heighten academic anxiety, weakening the coping resources required to rebound from everyday setbacks (Martin & Marsh, 2008). Consistent with this mechanism, prior work suggests that reduced buoyancy may be accompanied by lower academic engagement, and sustained disengagement can facilitate the consolidation of strain into burnout (Jia et al., 2020; Xu, 2023). In this sense, buoyancy serves as a psychologically meaningful resource-related process that helps explain how self-protective self-handicapping may be linked to later exhaustion and disengagement, although this pathway should be interpreted cautiously.

Meanwhile, the findings provided clearer support for a reverse indirect effect. Burnout is marked by exhaustion, alienation, and reduced accomplishment, which can erode students’ sense of control and motivational energy, thereby weakening buoyancy and making avoidant self-protective strategies more likely (Martin & Marsh, 2008; Schaufeli et al., 2002; Wang et al., 2025). When students experience lower burnout, they may be more able to invest time and effort in schoolwork, which can help sustain buoyancy (Jia et al., 2020); higher buoyancy, in turn, may facilitate more adaptive coping and reduce reliance on self-handicapping (Sun, 2023). Although this reverse indirect effect warrants further examination, together these findings underscore buoyancy as a central process variable in the longitudinal association between self-handicapping and burnout.

It is also noteworthy that the direct cross-lagged association between academic self-handicapping and academic burnout was asymmetric in the present model: academic self-handicapping did not significantly predict subsequent burnout, whereas academic burnout significantly predicted subsequent self-handicapping. One plausible explanation is that the longitudinal association from self-handicapping to burnout may operate primarily through intervening processes such as academic buoyancy, rather than through a direct cross-lagged association. In addition, after controlling for autoregressive stability in the CLPM, direct cross-lagged associations may be attenuated, particularly when their effects are relatively small (Adachi & Willoughby, 2015). Together, these considerations may help explain why the direct cross-lagged association between academic self-handicapping and academic burnout appeared asymmetric in the present model.

The relatively small effect sizes also require careful interpretation. Consistent with prior recommendations for interpreting longitudinal autoregressive models, small cross-lagged paths may still be meaningful when they are theoretically expected and replicated across adjacent time intervals (Adachi & Willoughby, 2015). In the present study, the practical significance of the findings lies less in the size of any single coefficient and more in the consistent pattern linking self-handicapping, reduced buoyancy, and burnout-related risk over time.

### 4.4. Research Limitations and Implications

Consistent with the mechanism-focused account proposed in the Introduction, the findings suggest that interventions targeting academic buoyancy may be especially useful. Although the present study advances understanding of how academic self-handicapping, academic buoyancy, and academic burnout are interrelated during early adolescence, two limitations should be acknowledged. First, the core constructs were measured primarily via adolescents’ self-reports, which may be influenced by response tendencies and subjective interpretations. Future research could strengthen measurement validity by integrating multi-informant and multi-method evidence, such as teacher or caregiver reports, behavioral indicators, and classroom-based observations (Podsakoff et al., 2003). Second, while a multi-wave design helps clarify temporal ordering, the observational nature of the data still limits causal inference. Future work could adopt intervention or experimental designs that directly strengthen buoyancy or reduce self-handicapping to more rigorously test the proposed mechanism and its boundary conditions (Adachi & Willoughby, 2015).

Despite these limitations, the findings offer cautious practical implications for school-based mental health and prevention in educational settings, particularly when understood from a cumulative prevention perspective. Given the small magnitude of the longitudinal effects, these implications should not be interpreted as evidence for large short-term changes, but rather as indicating potential targets for early identification and sustained preventive support. Schools may consider routine screening and tiered preventive support to identify students showing elevated self-handicapping or burnout and weakened buoyancy, enabling timely, targeted assistance. More specifically, screening could combine brief self-report measures with teacher observations of coping-related indicators, such as repeated procrastination, effort withdrawal, excuse-making after poor performance, reduced recovery after setbacks, exhaustion, and/or disengagement. Such screening could be conducted at key points in the school year, for example at the beginning of each semester or after major examinations, and students could then be allocated to different levels of support: universal classroom-level activities for all students, targeted small-group support for students showing moderate risk, and individualized counseling or referral for students with persistent high self-handicapping, low buoyancy, or severe burnout-related symptoms. At the same time, teacher professional development can focus on recognizing self-handicapping signals and responding with supportive, autonomy-respecting practices, while embedding brief “buoyancy micro-skills” into daily instruction, such as structured reflection after setbacks, planning next-step efforts, and rehearsing problem-focused coping routines (Martin & Marsh, 2008; Putwain et al., 2022). For example, teachers can reduce self-handicapping by reframing feedback away from fixed ability and toward controllable strategies, effort regulation, and next-step planning. Classroom climate may also be adjusted by normalizing academic setbacks, reducing shame-based comparison, and treating mistakes as information for improvement rather than as evidence of low ability. In everyday teaching, teachers can reinforce adaptive coping by asking students after quizzes, assignments, or examinations to identify one controllable reason for difficulty and one concrete action for the next attempt. In this context, buoyancy micro-skills may include brief setback reflection, emotion labeling, controllable attribution, help-seeking scripts, and problem-focused coping rehearsal. These skills can be taught through short prompts, reflection cards, or brief post-task routines and integrated into regular instruction across subjects, such as mathematics problem correction, language writing feedback, or science task review. Finally, optimizing evaluation and feedback practices toward more formative and mastery-oriented approaches may reduce performance-threat climates that often motivate self-protective avoidance and thereby help interrupt early burnout trajectories (Jia et al., 2020; Sun, 2023; Xu, 2023). Together, these strategies align with an early identification and tiered prevention logic by simultaneously reducing coping-related risk markers (self-handicapping) and strengthening protective resources (buoyancy).

## 5. Conclusions

(1) Academic self-handicapping and academic buoyancy showed a reciprocal negative longitudinal association. Specifically, T1 academic self-handicapping significantly negatively predicted T2 academic buoyancy, and T2 academic self-handicapping significantly negatively predicted T3 academic buoyancy; conversely, T1 academic buoyancy significantly negatively predicted T2 academic self-handicapping, and T2 academic buoyancy significantly negatively predicted T3 academic self-handicapping.
(2) Academic buoyancy and academic burnout showed a reciprocal negative longitudinal association. Specifically, T1 academic buoyancy significantly negatively predicted T2 academic burnout, and T2 academic buoyancy significantly negatively predicted T3 academic burnout; conversely, T1 academic burnout significantly negatively predicted T2 academic buoyancy, and T2 academic burnout significantly negatively predicted T3 academic buoyancy.
(3) Academic buoyancy showed a nuanced longitudinal mediating pattern. Specifically, the hypothesized indirect effect from academic self-handicapping to academic burnout via academic buoyancy was marginally significant, whereas the reverse indirect effect from academic burnout to academic self-handicapping via academic buoyancy was significant.

## Figures and Tables

**Table 1:** Longitudinal Measurement Equivalence Tests for Academic Self-Handicapping, Academic Buoyancy, and Academic Burnout.

Variable	Model	χ^2^	df	CFI	RMSEA	∆CFI	∆RMSEA
Academic Self-Handicapping	Configuration equivalence	1956.37	891	0.908	0.049		
	Weak equivalence	2022.81	919	0.905	0.049	−0.003	0.000
	Strong equivalence	2192.81	947	0.892	0.051	−0.013	0.001
Academic Buoyancy	Configuration equivalence	72.194	39	0.984	0.041		
	Weak equivalence	86.630	45	0.980	0.043	−0.004	0.002
	Strong equivalence	112.231	51	0.970	0.049	−0.010	0.006
Academic Burnout	Configuration equivalence	1946.76	954	0.916	0.045		
	Weak equivalence	2023.90	985	0.912	0.046	−0.004	0.001
	Strong equivalence	2152.46	1013	0.904	0.047	−0.008	0.001

**Table 2:** Descriptive Statistics and Correlation Analysis of Variables (n = 508).

	M	SD	1	2	3	4	5	6	7	8	9
1	1.685	0.506	1								
2	1.732	0.609	0.564 **	1							
3	1.743	0.676	0.400 **	0.533 **	1						
4	4.261	1.135	−0.373 **	−0.341 **	−0.218 **	1					
5	4.233	1.161	−0.257 **	−0.309 **	−0.261 **	0.591 **	1				
6	4.184	1.006	−0.331 **	−0.354 **	−0.302 **	0.480 **	0.581 **	1			
7	2.834	0.477	0.384 **	0.350 **	0.232 **	−0.510 **	−0.432 **	−0.378 **	1		
8	2.893	0.466	0.384 **	0.394 **	0.319 **	−0.458 **	−0.429 **	−0.402 **	0.757 **	1	
9	2.878	0.464	0.278 **	0.314 **	0.334 **	−0.360 **	−0.406 **	−0.486 **	0.620 **	0.700 **	1

Note. T1, T2, and T3 denote the first, second, and third measurement waves, respectively. Variable codes are as follows: 1 = academic self-handicapping at T1, 2 = academic self-handicapping at T2, 3 = academic self-handicapping at T3; 4 = academic buoyancy at T1, 5 = academic buoyancy at T2, 6 = academic buoyancy at T3; 7 = academic burnout at T1, 8 = academic burnout at T2, and 9 = academic burnout at T3; ** *p* < 0.01. Values reported in the text are rounded to two decimal places for readability.

**Table 3:** Key Standardized Cross-Lagged Paths and Indirect Effects in the Final Model.

Research Question	Effect	Estimate	*p*	95% CI
H1	ASH T1 → AB T2	−0.085	<0.01	—
H1	ASH T2 → AB T3	−0.087	<0.01	—
H1	AB T1 → ASH T2	−0.098	<0.01	—
H1	AB T2 → ASH T3	−0.100	<0.01	—
H2	AB T1 → BO T2	−0.096	<0.01	—
H2	AB T2 → BO T3	−0.097	<0.01	—
H2	BO T1 → AB T2	−0.141	<0.001	—
H2	BO T2 → AB T3	−0.143	<0.001	—
H3	ASH T1 → AB T2 → BO T3	0.008	0.059	[0.000, 0.017]
Reverse indirect effect	BO T1 → AB T2 → ASH T3	0.014	0.014	[0.003, 0.025]

Note. ASH = academic self-handicapping; AB = academic buoyancy; BO = academic burnout. Estimates for cross-lagged paths are standardized path coefficients, denoted as *γ* in the text. Estimates for indirect effects are standardized indirect effects based on the STDYX standardized results from Mplus. Confidence intervals are reported for indirect effects. “—” indicates that confidence intervals were not reported for individual cross-lagged paths.

## Data Availability

The data that support the findings of this study are available from the corresponding author upon reasonable request. The data are not publicly available because the dataset contains information from minor participants and is subject to privacy and ethical protection requirements.
